# Assessing secondary attack rates among household contacts at the beginning of the influenza A (H1N1) pandemic in Ontario, Canada, April-June 2009: A prospective, observational study

**DOI:** 10.1186/1471-2458-11-234

**Published:** 2011-04-14

**Authors:** Rachel Savage, Michael Whelan, Ian Johnson, Elizabeth Rea, Marie LaFreniere, Laura C Rosella, Freda Lam, Tina Badiani, Anne-Luise Winter, Deborah J Carr, Crystal Frenette, Maureen Horn, Kathleen Dooling, Monali Varia, Anne-Marie Holt, Vidya Sunil, Catherine Grift, Eleanor Paget, Michael King, John Barbaro, Natasha S Crowcroft

**Affiliations:** 1 Ontario Agency for Health Protection and Promotion, Toronto, Ontario, Canada; 2 Ontario Ministry of Health and Long-Term Care, Toronto, Ontario, Canada; 3 Toronto Public Health, Toronto, Ontario, Canada; 4 York Region Health Department, Newmarket, Ontario, Canada; 5 Oxford County Public Health and Emergency Services, Woodstock, Ontario, Canada; 6 Peel Public Health, Mississauga, Ontario, Canada; 7 Haliburton, Kawartha, Pine Ridge District Health Unit, Port Hope, Ontario, Canada; 8 Middlesex-London Health Unit, London, Ontario, Canada; 9 Sudbury & District Health Unit, Sudbury, Ontario, Canada; 10 Simcoe Muskoka District Health Unit, Barrie, Ontario, Canada; 11 Dalla Lana School of Public Health, University of Toronto, Toronto, Ontario, Canada

## Abstract

**Background:**

Understanding transmission dynamics of the pandemic influenza A (H1N1) virus in various exposure settings and determining whether transmissibility differed from seasonal influenza viruses was a priority for decision making on mitigation strategies at the beginning of the pandemic. The objective of this study was to estimate household secondary attack rates for pandemic influenza in a susceptible population where control measures had yet to be implemented.

**Methods:**

All Ontario local health units were invited to participate; seven health units volunteered. For all laboratory-confirmed cases reported between April 24 and June 18, 2009, participating health units performed contact tracing to detect secondary cases among household contacts. In total, 87 cases and 266 household contacts were included in this study. Secondary cases were defined as any household member with new onset of acute respiratory illness (fever or two or more respiratory symptoms) or influenza-like illness (fever plus one additional respiratory symptom). Attack rates were estimated using both case definitions.

**Results:**

Secondary attack rates were estimated at 10.3% (95% CI 6.8-14.7) for secondary cases with influenza-like illness and 20.2% (95% CI 15.4-25.6) for secondary cases with acute respiratory illness. For both case definitions, attack rates were significantly higher in children under 16 years than adults (25.4% and 42.4% compared to 7.6% and 17.2%). The median time between symptom onset in the primary case and the secondary case was estimated at 3.0 days.

**Conclusions:**

Secondary attack rates for pandemic influenza A (H1N1) were comparable to seasonal influenza estimates suggesting similarities in transmission. High secondary attack rates in children provide additional support for increased susceptibility to infection.

## Background

Upon detection of a novel strain of influenza A (H1N1) virus in California and Mexico in March 2009 and subsequent global distribution, the World Health Organization declared an influenza pandemic on June 11, 2009 [1]. Understanding the transmission dynamics of this novel virus was quickly deemed a priority in order to develop effective mitigation strategies which would minimize transmission until a vaccine was available. Recommended control measures for seasonal influenza epidemics include influenza vaccination, hand hygiene and cough/sneeze etiquette, environmental cleaning and self-isolation; however, pandemic plans also consider travel restrictions, mass immunization, school closures and restriction of mass public gatherings as potentially effective mitigation strategies [2]. To determine which control measures to recommend and how these measures should be targeted to populations at highest risk, estimation of pandemic (H1N1) 2009 (pH1N1) transmission risk in various exposure settings and population subgroups was necessary.

While factors influencing pH1N1 transmission were largely unknown at the beginning of the pandemic, seasonal influenza transmission studies had demonstrated the importance of household settings and young children in disease propagation. Transmission modes for seasonal influenza include direct contact with infected individuals, indirect contact with contaminated objects, and inhalation of droplet particles released through coughing and sneezing of infected individuals [3]. Given the importance of close contact or shared environment with infected individuals, households are important exposure settings for influenza transmission. Simulation models have estimated 30% to 40% of influenza transmission occurs in households compared to 20% in schools and the remainder in community settings [4]. Similarly, the role of children in influenza transmission has been demonstrated by Loeb *et al *(2010) who found that immunizing approximately 80% of children with trivalent influenza vaccine conferred 61% indirect protection for unimmunized residents of Hutterite communities in Western Canada [5].

In anticipation of the pandemic, the provincial Ministry responsible for the pandemic response, the Ontario Ministry of Health and Long-Term Care (MOHLTC), issued an enhanced surveillance directive to local health units requesting the investigation of severe and febrile respiratory illness cases and entry of mandatory data elements into the provincial information system used for reporting case information on reportable communicable diseases to the MOHLTC. All laboratory-confirmed cases of pH1N1 identified prior to June 20, 2009 were investigated by health units and additionally, seven health units volunteered to perform contact tracing in household settings during the first nine weeks of pandemic virus circulation in Ontario to provide estimates of transmissibility. The objectives of this study were to estimate secondary attack rates of pH1N1 in a susceptible population where additional control measures, such as mass immunization and antiviral prophylaxis, had yet to be implemented and to identify population subgroups at highest risk for illness.

## Methods

### Setting

Ontario's public health system consists of 36 local health units responsible for the delivery of local public health programs, one central and 11 regional public health laboratories.

### Data Collection

The Ontario Agency for Health Protection and Promotion (OAHPP) Laboratory (Toronto) performed the majority of pH1N1 testing in Ontario; 84% of pH1N1 cases detected during the first wave (April 19, 2009 - August 29, 2009) were tested at OAHPP and 69% in the second wave (August 30, 2009 - January 31, 2010). All respiratory specimens submitted to OAHPP laboratories were tested. Specimens were initially tested using a combination of endpoint reverse transcriptase (RT) polymerase chain reaction (PCR) for the influenza A virus matrix gene and sequencing. Specimens submitted after May 15, 2009 were tested using a more sensitive real-time RT-PCR for the pH1N1 (H and N genes) developed at the Toronto laboratory. The Luminex Respiratory Virus Panel multiplex assay and viral culture were used to test approximately 6% of specimens [6].

While specimens were triaged so that patients at high risk for complications from influenza were prioritized, testing practices and triaging remained consistent with one exception during this study period. Initially, patients in emergency departments and ambulatory care settings presenting with severe and febrile respiratory illness and who reported travel to Mexico were recommended for testing; travel criteria were removed on May 19, however, once sustained community spread was established. Testing was later restricted to patients at high risk for complications from influenza; although, this did not occur until the end of the study period. Laboratory-confirmed cases of pH1N1 were reported to local health units who performed detailed case investigations using a standardized case report form. Case information was entered into the integrated Public Health Information System (iPHIS), the information system used by Ontario's 36 public health units for reporting case information on reportable communicable diseases to the MOHLTC.

In total, 7/36 (19%) health units volunteered to participate in this study. Staff at these health units performed telephone interviews with index cases reported in iPHIS between April 24 and June 18, 2009 to collect case information and identify household contacts. This nine-week period spanned the time to (and included) the peak of the first wave in Ontario [7]. For two health units with case volumes of >100, contact tracing was restricted to cases reported up to May 24, 2009. Information regarding self-reported exposure dates, symptoms, symptom onset, illness duration, specimen testing, hospitalization status, and 2008/9 seasonal influenza immunization status of contacts was collected using a standard questionnaire. Additionally, laboratory testing for symptomatic contacts was encouraged but did not occur in most cases. Household contact information was linked to case records through their iPHIS unique identifier. Using this approach, all records were successfully linked.

Primary cases were defined as household members with the earliest symptom onset date and laboratory-confirmation of pandemic influenza A (H1N1) virus infection by one or more of the following laboratory tests: RT-PCR with genotyping of H1 and/or N1 novel influenza virus, viral culture with strain typing or four-fold rise in novel influenza A (H1N1) virus specific antibodies by serology testing, or as the first person with influenza-like illness (fever and ≥1 respiratory symptom such as cough, sore throat, arthralgia, myalgia and malaise) when another household member had a laboratory-confirmed pH1N1 infection. Household contacts were defined as persons who had close contact (≥1 hour exposure within 2 metres) with a laboratory-confirmed case in a household setting (shared, common accommodation in terms of both sleeping and eating at least one meal). All primary cases were eligible for inclusion in this study if ≥1 household contact was identified. Secondary cases were defined either as household contacts with acute respiratory illness (ARI, fever or ≥2 respiratory symptoms) or influenza-like illness (ILI), with symptom onset >1 day to ≤14 days following symptom onset of the primary case. Secondary household attack rates were calculated as the proportion of household contacts, excluding the primary case, meeting either secondary case definition.

Due to workload and other logistical concerns, primary case follow-up by public health units occurred at different times. Where initial case contact occurred ≥7 days after symptom onset, the primary case was only contacted once and all relevant information on the case and household contacts was obtained. If the initial investigation was conducted <7 days after symptom onset, the primary case was re-contacted a second time ≥7 days after symptom onset, to further investigate whether any new symptomatic contacts had developed. Data on the investigation timeframe was collected in order to detect potential ascertainment bias.

### Analysis

Household contacts and the associated index case were excluded from analysis if the contacts were lost to follow-up or untraceable. If the serial interval, defined as the time between symptom onset in the primary case and the secondary case, was ≤1 day, the household contact was defined as a co-primary case and excluded from secondary attack rate calculations. Symptomatic contacts with an onset date preceding the case onset date by 2-5 days that met the ILI case definition or tested positive for pH1N1 were reclassified as primary cases.

The 95% confidence intervals (CI) for secondary household attack rates were calculated from the binomial distribution using exact methods [8]. Secondary attack rates were cumulative and likely included tertiary cases; however, secondary cases with a serial interval of >14 days from the primary case were excluded in serial interval and secondary attack rate calculations.

To test for differences between cases and contacts, Pearson chi-squared and Wilcoxon rank sum tests were used for categorical and continuous variables, respectively. The statistical analysis was conducted using STATA Version 10.1 (Statacorp, Texas, USA).

This study was conducted as a legally mandated public health investigation under the authority of the Ontario Health Protection and Promotion Act (R.S.O. 1990, c.H.7).

## Results

In total, 103 laboratory-confirmed index cases were interviewed by public health staff, identifying 290 household contacts. Ten cases and their nine contacts (4.8%) were excluded because household contacts were either not identified or lost to follow-up. Additionally, 6 index cases who did not meet the primary case definition were excluded along with their 15 contacts (5.3%). Two contacts had onset dates which preceded the primary case by 3 days and were reclassified as primary cases, leaving 87 laboratory-confirmed cases of pH1N1 and 266 household contacts included in this analysis.

A mean of 3 household contacts were identified per primary case (range 1-7). Cases were followed up, on average, 8 days after symptom onset (range 1 - 22 days). For 79 cases (90.8%), investigations were completed ≥7 days after symptom onset. In total, 51 secondary cases were identified in 34/87 (39.1%) households; 5 households each with 2 secondary cases, 3 households with 3 secondary cases and 2 households with 4 secondary cases.

### Characteristics of primary cases and household contacts

Cases were significantly younger than their household contacts (median difference = 16 years, p = 0.005); age was missing for 49 (18.4%) household contacts (Table 1). Forty-seven cases (54.0%) and 119 contacts (44.7%) were female (sex unknown for 14 contacts). Secondary cases had a similar median age and gender distribution as primary cases. Sixteen cases (18.4%), 73 contacts (27.4%) and 15 (29.4%) secondary cases reported receiving the 2008/9 seasonal influenza vaccine; however, missing vaccination status was more likely for contacts (28.6%) and secondary cases (25.5%) compared to cases (9.2%). The majority of cases (85.1%) reported experiencing ILI compared to 11.7% of household contacts and 51.0% of secondary cases. One case was hospitalized for 7 days as a result of pH1N1 infection. There were no known deaths.

**Table 1:** Characteristics of primary cases and their household contacts.

Characteristic	Primary Cases n (%)	Household Contacts n (%)	Secondary Cases n (%)
Sex, Female	47 (54.0)	119 (44.7)	25 (49.0)
Age, median (range)	18 yrs (1-66)	34 yrs (1-81)	16 yrs (1-62)
Under 16 yrs	34 (39.1)	64 (24.1)	25 (49.0)
2008/9 Seasonal Influenza Vaccination	16 (18.4)	73 (27.4)	15 (29.4)
Reported ARI	85 (97.7)	64 (24.1)	51 (100.0)
Reported ILI	74 (85.1)	31 (11.7)	26 (51.0)
Reported Diarrhea or Vomiting*	21 (24.1)	5 (1.9)	5 (9.8)
Hospitalization	1 (1.1)	0 (0.0)	0 (0.0)
Specimen submitted and tested for pH1N1	86 (98.9)	28 (10.5)	17 (33.3)

	N = 87	N = 266	N = 51

ARI, acute respiratory illness. ILI, influenza-like illness.

* Specific symptoms were not explicitly asked of contacts

### Serial Interval

**Figure 1:**
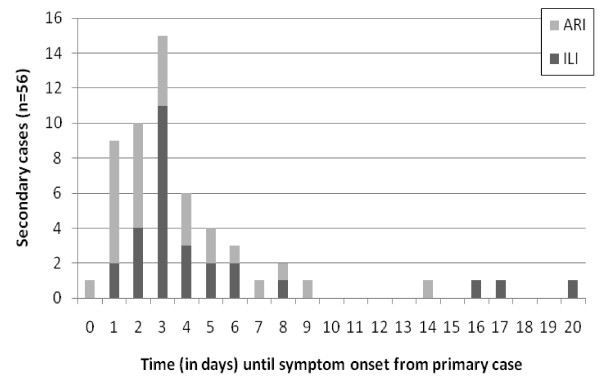
**Distribution of time from symptom onset of primary case to symptom onset of secondary case**. Note: Co-primary cases are included. Date of onset was unknown for 8 secondary cases. ARI, acute respiratory illness. ILI, influenza-like illness.

In total, 64 household contacts (including co-primary and tertiary cases) met either the ARI or ILI secondary case definition. Symptom onset dates were known for 56 contacts and their 39 primary cases. The median serial interval was estimated at 3.0 days (range 0 - 20 days) (Figure 1); there was no difference in median serial interval for symptomatic contacts with ILI compared to those with ARI.

### Secondary Attack Rates

Ten contacts reported a symptom onset date within 1 day of the primary case onset date and were reclassified as co-primary cases, resulting in 97 primary/co-primary cases and 256 contacts. Three symptomatic contacts with a serial interval >14 days were excluded from this analysis; secondary attack rates (SARs) were calculated among the remaining 253 contacts (Table 2). SARs were estimated at 10.3% (95% CI 6.8-14.7) for secondary cases with ILI and 20.2% (95% CI 15.4-25.6) for secondary cases with ARI. Rates were significantly higher in children than adults (p = 0.001 and p < 0.001). When stratified further by age, SARs were highest in contacts aged 5-15 years (15/51 or 29.4% for ILI and 23/51 or 45.1% for ARI) and 25-39 years (5/29 or 17.2% for ILI and 8/29 or 27.6% for ARI). Rates were lowest in contacts aged 16-24 years (1/27 or 3.7% for ILI and 3/27 or 11.1% for ARI) and 65 years and above (0/8 or 0.0% for ILI and 0/8 or 0% for ARI) (data not shown). Of all contacts with unknown age (n = 49), only 1 met the ARI case definition; the remainder did not report experiencing respiratory symptoms. Excluding all households with co-primary cases (n = 8) did not change SAR estimates.

**Table 2:** Household secondary attack rates by secondary case definition and age group.

Secondary Case Definition	Age Group	Contacts (N)	Secondary Cases	SAR (%)	95% CI	P-value*
ARI	Overall	253	51	20.2	15.4 - 25.6	
	Children <16 years	59	25	42.4	29.6 - 55.9	<0.001
	Adults ≥16 years	145	25	17.2	11.5 - 24.3	

ILI	Overall	253	26	10.3	6.8 - 14.7	
	Children <16 years	59	15	25.4	15.0 - 38.4	0.001
	Adults ≥16 years	145	11	7.6	3.8 - 13.2	

Note: Age was missing for 49 contacts.

ARI, acute respiratory illness. ILI, influenza-like illness. CI, confidence interval.

*Pearson chi-squared test for the difference in SAR between children and adults.

### Description of Laboratory Tested Contacts

Nasopharyngeal swabs were submitted and tested for 28 household contacts. Twelve tested positive for pH1N1 (42.9%), 1 result was indeterminate, and 2 tested positive for seasonal influenza A (7.1%); the remaining 13 tests were negative. Among those persons who tested positive for pH1N1, 11/12 (91.7%) experienced ILI, compared to 7/16 (43.8%) of the negative pH1N1 contacts (p = 0.009). Duration of illness was recorded for 5 positive pH1N1 contacts and 6 negative or indeterminate pH1N1 contacts. The median duration of illness was longer among contacts testing positive for pH1N1 (8 days, range 2-11) compared to test negatives or indeterminates (3.5 days, range 1-9 days); although, this difference was not statistically significant (p = 0.196).

Specimen collection date was available for all 28 contacts. For contacts on whom a specimen was collected within 7 days of symptom onset, the median difference in time between symptom onset and specimen collection was 1 day for positive pH1N1 contacts and 2.5 days for negative/indeterminate pH1N1 contacts (p = 0.272).

## Discussion

This study reports on household follow-up of 87 laboratory-confirmed pH1N1 cases in Ontario from late April to early June. Overall household secondary attack rates (SARs) were estimated between 10.3% and 20.2%, with higher rates observed in children compared to adults. These rates largely reflect transmission in the absence of pharmaceutical intervention and therefore provide a pure estimate which more closely characterizes transmission dynamics without the influence of intervening measures. Although not asked in this study, household contacts were unlikely to have received antiviral prophylaxis as this public health measure was not recommended in Ontario [9] and the provincial stockpile of antivirals was not released until October 22, 2009 [10]. In fact, despite the recommendation that antivirals be prescribed for patients with ILI at high risk of complications, in an Ontario study examining risk factors for pH1N1 infection, only 20% of laboratory-confirmed cases of pH1N1 reported receiving antiviral treatment during the first wave (personal communication, Laura Rosella).

Results from this study corroborate two published pH1N1 transmission studies in Japan and California where SARs of 7.6% and 6% were estimated among household contacts who did not receive antiviral prophylaxis [11,12]. Similarly, an analysis of household transmission of pH1N1 across the United States (U.S.) found that 13% of household contacts of laboratory-confirmed cases reported ARI and 10% reported ILI; information on antiviral prophylaxis of household contacts was not available [13]. Higher household SARs for pH1N1 have been reported in several studies [4,14-17]. Two of these reports, however, were based on follow up of one index case in non-household settings (student and tour groups) where interactions between individuals may differ from traditional households, while another two studies performed daily follow-up of households and for a longer duration of time (8 and 14 days).

Household transmission studies for seasonal influenza have reported SARs ranging from 5% to 40%, suggesting similarities in transmission dynamics to pH1N1 [18-23]. The broad range of seasonal influenza SARs and influenza subtypes, however, makes direct comparison difficult.

Although we were unable to examine multiple risk factors for infection, this study demonstrated higher SARs for children; a finding consistent with previous studies [24,25]. Age-related differences in infection may be explained by increased susceptibility of children to pH1N1 infection due to lack of pre-existing immunity [26,27], increased contact with a larger number of extra-household contacts than adults through school/daycare [22], differing contact behaviour within the household and poor adherence to personal protective measures such as hand washing.

At an ecological level, hypotheses for additional risk factors can be generated through comparison of SARs across regions where differing public health interventions were applied. In the U.S. and United Kingdom (U.K.), policies on antiviral prophylaxis differed from Ontario. While the broad use of antiviral prophylaxis was not recommended, the policies stated that antiviral prophylaxis could be considered for close contacts of suspected or laboratory-confirmed cases at high risk of developing complications of influenza, health care workers and emergency medical personnel and pregnant women [28,29]. In the U.K., SARs of 18.9% for secondary contacts with ARI, 10.5% for contacts with ILI and 8.1% for contacts with laboratory confirmed pH1N1 were reported, with 50% of contacts reporting receipt of antiviral prophylaxis four days after onset of illness in the index case [30]. Additionally, two household transmission studies in New York City and San Antonio report comparable SARs (9% and 13%), although, uptake of antiviral prophylaxis in household contacts was low [25,24]. While all studies demonstrate significant reductions in transmission within households where antiviral prophylaxis was administered, the overall impact of these policies on transmission at a population level remains unknown.

Comparable attack rates despite differing interventions may be explained by the index case population included in our study. Nishiura *et al *(2010) have demonstrated the impact of initial conditions on estimating transmissibility of an emerging pathogen; in Japan, the estimated reproduction number for late May to early July was much smaller than that estimated at the beginning of the pandemic when transmission was confined mainly to school settings [31]. In this study we did not have data from health units with a large number of school outbreaks. By not capturing a similar proportion of the school-aged cases that played a major role in the early spread of the pandemic virus, we may have underestimated overall transmission rates in the population.

Bias introduced through case ascertainment may have also underestimated SARs. Clinical case definitions cannot capture all mild or asymptomatic cases; serological testing is the most sensitive method for detecting incident cases. One published study on pH1N1 transmission in Quebec City, Canada performed systematic serial laboratory testing (including serological testing) to evaluate household contacts. Using this approach, Papenburg *et al *(2010) found a household SAR of 45% for contacts with laboratory-confirmed pH1N1, which is considerably higher than rates reported in this study despite similar policies on antiviral prophylaxis and than other published studies reliant on clinical syndromes to define secondary cases [32]. Incomplete case follow-up may have similarly underestimated SARs. While health units were requested in this study to monitor households for a minimum of seven days, 9.2% of cases had a shorter follow-up period. Despite this, if we missed 30% of true secondary cases, the SAR for contacts with ILI would have increased by 3% from 10.3% to 13.4%, which still lies within ranges reported by other studies.

Cases with milder disease were captured in this study through broader, clinical case definitions. While these definitions are more sensitive, they are less specific and can include cases without pH1N1 infection. During the study period, entero- and rhinoviruses were co-circulating and could have been the causative organism for some secondary cases; however, these viruses were not identified in any laboratory tested contacts. Seasonal influenza virus was also circulating in Ontario at this time and detected in approximately 5% of all patients tested at the OAHPP laboratories from late April until early June, as well as 2 of the 28 contacts in this study [33]. This finding illustrates the limitation of using clinical syndromes, and not laboratory testing, to define cases. Lastly, by the end of May 2009 in Ontario, pH1N1 was endemic in the community; consequently, secondary cases may have been misattributed to the household primary case when acquisition occurred outside of the home, which would similarly inflate SARs.

## Conclusions

Despite these limitations, the transmission characteristics estimated by this study are in alignment with other published reports and offer the added value of estimating transmission risk in a population where public health interventions, such as antiviral prophylaxis and school closures, were not yet implemented. Moderate household secondary attack rates demonstrate the importance of close contact among household members in the transmission of pH1N1, while high secondary attack rates in children provide additional support for increased susceptibility to pH1N1 infection. We believe that the findings of this study will be of interest to a wide audience of public health physicians, clinicians, modelers and policy makers who are involved in influenza and pandemic planning and response. Given the limitations of clinical definitions for case ascertainment, other methods, such as serological testing in a prospective study, should be employed in future to determine the effectiveness of intervening public health measures in reducing transmission and to understand the risk of transmission in various exposure settings.

## Competing interests

The authors declare that they have no competing interests.

## Authors' contributions

RS participated in the study's design and coordination, performed the statistical analysis, and drafted the manuscript. MW participated in the study's design and coordination, and helped to perform the statistical analysis and draft the manuscript. IJ and ER conceived of the study, and participated in its design. ML assisted in the statistical analysis. LR assisted in the statistical analysis, and helped to draft the manuscript. TB and AW participated in the study's design. FL, DJC, CF, MH, KD, MV, AH, VS, CG, EP, MK, JB participated in data collection and interpretation of the data. NSC conceived of the study, participated in its design, and helped to perform the statistical analysis and draft the manuscript. All authors read and approved the final manuscript.

## Pre-publication history

The pre-publication history for this paper can be accessed here:

http://www.biomedcentral.com/1471-2458/11/234/prepub
